# The Potential Use of P1 CAEP as a Biomarker for Assessing Central Auditory Pathway Maturation in Hearing loss and Associated Disabilities: a case report

**DOI:** 10.25122/jml-2019-0096

**Published:** 2019

**Authors:** Cristina Pantelemon, Violeta Necula, Livia Livint Popa, Steluta Palade, Stefan Strilciuc, Dafin Fior Muresanu

**Affiliations:** 1. Department of Neurosciences, “Iuliu Hatieganu” University of Medicine and Pharmacy, Cluj-Napoca, Romania; 2. “RoNeuro” Institute for Neurological Research and Diagnostic, Cluj-Napoca, Romania; 3. Department of ENT, “Iuliu Hatieganu” University of Medicine and Pharmacy, Cluj-Napoca, Romania; 4. Department of Pediatric Neurology, Children’s Emergency Hospital Cluj-Napoca, Cluj-Napoca, Romania; 5. Department of Public Health, Babes-Bolyai University, Cluj-Napoca, Romania

**Keywords:** hearing loss, auditory cortex, auditory pathway, P1 component of CAEP

## Abstract

We report a case in which we quantified the maturation of the central auditory pathway in children with hearing loss and associated disabilities; the audiological intervention was performed using the BAHA softband. The hearing aid was applied according to the international clinical protocols. The presented case reveals the importance of using the P1 CAEP biomarker in clinical practice along with a neuropsychological evaluation to assess the maturation of the central auditory pathways and to objectively quantify the results of auditory rehabilitation in children with hearing loss and associated disabilities.

## Introduction

Hearing loss, especially in children, may cause a delay in language development, and consequently, it may affect cognition and lead to learning disabilities [1]. For this reason, the process of auditory rehabilitation must begin early. Early auditory stimulation allows for the appropriate maturation of central auditory pathways, which is necessary for the further development of language. By analyzing the morphology and latency of the P1 component, cortical auditory evoked potentials (CAEPs) represent an objective method of quantifying the maturation of the auditory cortex [2]. In children with a normal hearing, the average latency of the P1 wave is approximately 300 ms. In the first years of life, there is a significant drop in latency, so that by around the time the child is three years old, latency is 125 ms, and later on, in the adult stage, it gets to 60 ms [3]. The decrease in latency of the P1 wave reflects the modifications at the level of synaptic connections and the synchronization of neuronal transmission at the level of the central auditory pathways [4]. By the time the child reaches 3.5 years of age, the auditory cortex has increased plasticity that will, later on, decrease considerably. Therefore, if auditory rehabilitation is done during this time, the latency of the P1 wave drops and reaches the normal limits [2].

In the case of the auditory cortex not receiving auditory stimuli in an optimal period, then we are dealing with the phenomenon of cross reorganization (auditory areas develop visual and somatosensorial functions) [5-7]. 30-40% of children with hearing loss have multiple associated disabling conditions: psychomotor delay, visual impairment, cognitive impairment, language disorder, brain structural changes, and behavioral problems [8].

The P1 CAEP biomarker is easy to record, non-invasive, and it is an objective parameter for assessing the efficacy of hearing aid use in children with hearing loss and associated disabilities [8].

## Case presentation

We present the case of a 38-month-old girl who was diagnosed with Goldenhar syndrome as a newborn. She was born at term following a normal pregnancy (birth weight = 3220 gr, APGAR score 8). Postnatally, she presented nasal obstruction, inspiratory dyspnea, feeding difficulties, vomiting after a feed, episodes of apnea and cyanosis after regurgitation of gastric contents. An ENT consultation was performed, and bilateral choanal atresia was identified; the surgical lysis of the imperforation was performed with stent mounting. The patient presented other malformations: microretrognathism, microcrania (head circumference > 2 standard deviations below the mean for gestational age), mandibular hypoplasia, low ear implantation, pre-auricular appendix, external auditory canal (EAC) atresia of the left ear, short neck, bilateral microtia. Brain computed tomography performed at 23 months revealed a cleft palate in the right parasagittal plane, adenoid hyperplasia with partial choanal atresia, left EAC atresia, right EAC stenosis; also, the left ossicle chain was not identified (excepting a piece of the hammer), while the internal auditory canal (IAC) had normal morphology. At 11 months, the diagnosis of moderate conductive/mixed hearing loss in the right ear and severe mixed hearing loss in the left ear was established (Table 1).

**Table 1:** Audiologic profile.

	Right ear (RE)	Left ear (LE)
**ABR – V wave present at**	80 dB HL	60 dB HL
**ASSR**	80 and 90 dB HL	60 and 70 dB HL
**Tympanogram**	type B	
**DPOAE**	refer	

ABR - Auditory brainstem responses, ASSR - Auditory steady-state responses, DPOAE - Distortion Product Otoacoustic Emissions, RE - right ear, LE - left ear.

Hearing rehabilitation was achieved using the BAHA Softband. The control audiogram at 38 months showed hearing thresholds at 30 dB HL in the sound field.

Cortical auditory potentials were recorded at 38 months to see if the amplification made by the BAHA 5 Softband hearing aid ensured proper maturation of the central auditory pathway. Testing was carried out within 90 minutes in an anechoic room. The patient was comfortably placed in the parent’s lap and watched cartoons on mute/silent mode. The electrodes were placed on the scalp as follows: the active Cz electrode connected to the positive input of the amplifier, the reference electrode on the mastoid process, and the ground electrode at Fpz. A supraorbital electrode, paired with an infraorbital reference electrode placed ipsilaterally, was used to minimize ocular artifacts. Electrode impedance was maintained between 1-3 kOhms. A calibrated loudspeaker placed at 1 m distance in 0° angle emitted a speech stimulus, the “ba” syllable, at 70 and 80 dB nHL intensity. The stimulus rate was 1.10/s, the duration was 114875 µsec, for 512 sweeps, artifact rejection with amplitude criterion at ± 100 µV. CAEPs recorded following the delivery of the stimulus were analyzed using the SmartEP USB software provided by Intelligent Hearing System.

**Figure 1:**
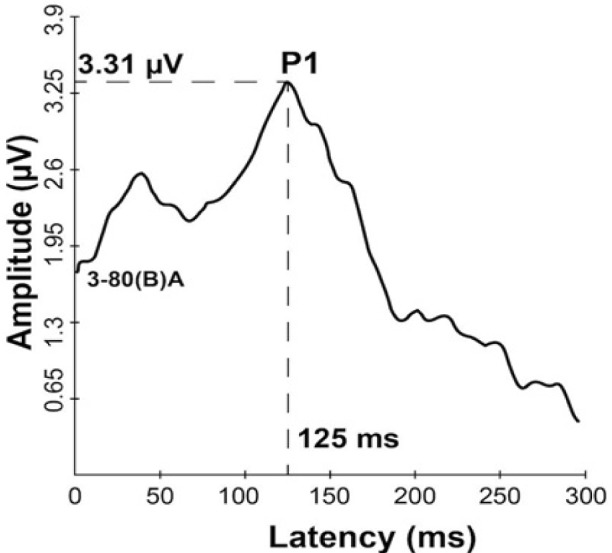
Grand average CAEP response after hearing aid intervention.

The measurement of the P1 CAEP biomarker after 27 months of hearing aid use shows normal latency (125 ms), morphology, and amplitude of 3.31μV (Figure 1). This finding indicates that the hearing aid performed adequate amplification, suggestive of normal central auditory pathway development.

We evaluated the patient using the Denver Developmental Screening Test II (DDST II) adapted for children with multiple disabilities. DDST II consists of 125 items that aim to evaluate the child in the following areas: personal-social, fine motor-adaptive, language, gross motor ability. It also includes five “behavior-during-testing” items that help the physician assess the child’s general behavior, obtaining a rough indicator of her abilities. The child’s performance is usually classified in one of three categories: “normal”, “suspect” or “untested”. The traditional scoring does not provide a quantitative estimation of development. In contrast, DDST II is specifically designed to detect varying degrees of developmental impairment, monitoring progress over time and child’s integration into specialized rehabilitation programs.

Developmental testing was first performed at 32 months using the Denver Developmental Screening Test II. Subsequently, after the patient was integrated into a rehabilitation program (physical therapy, speech therapy, and auditory-verbal stimulation), reevaluation was performed at 38 months (Table 2).

**Table 2:** Denver Developmental Screening Test II – conventional and adapted evaluation scales.

	Age (months)	Personal - Social	Fine motor - adaptive	Language	Gross motor	Result	DFE	Q
**Conventional (score)**	32	4D, 2C	2D, 1C	14D	1D, 2C	Suspect	n/a	n/a
	38	1D, 2C	1C	6D, 1C	2C	Suspect	n/a	n/a
**Adapted (months)**	32	15	17	9	16	n/a	14	0.43
	38	25	28	21	28	n/a	26	0.66

D = delay; C = caution; DFE = developmental functioning estimate; Q = developmental quotient score; GS = developmental gain score.

## Discussion

Our research is one of the few documented studies in which maturation of the central auditory pathway was evaluated in children with BAHA interventions.

In our patient, the therapeutic intervention to compensate for the auditory deficit was performed early, and this allowed the central auditory pathway to develop according to her age – P1 morphology and latency proper for age [9]. P1 can also be used as an objective indicator by which we can separate the effects of sensory deprivation from cognitive deficits in those children with associated disabilities. Thus, rehabilitation therapy can focus more on the cognitive aspects of language acquisition.

Pre- and post-intervention progress monitoring can be a difficult task for the clinician. In many cases, it is difficult to obtain objective measurements of auditory thresholds in children with multiple disabilities through audiograms with visual reinforcement because the child cannot be conditioned on the stimulus and the assessment of the perceptual language is limited (these children do not acquire open-set and close-set word recognition) [8].

In a study performed on 146 children aged 6 to 15 with a non-syndromic cleft palate or orofacial cleft, without hearing loss, Xiaoran Ma et al. demonstrated that P1-N1 amplitude was significantly decreased, and N1 had a higher latency compared to healthy children of the same age [10]. These results indicate a low auditory processing disorder and a delayed maturation of the central auditory pathway. The associated auditory disorder may be due to delayed myelination and synaptogenesis. Also, the hearing disorder in these children with cleft lip and cleft palate, or cleft palate is another factor that facilitates language and learning disorders [10]. In a different study performed on 46 male subjects who ranged in age from 18-47 years diagnosed with non-syndromic cleft lip and cleft palate or left palate, Noupoulus et al. identified imaging changes in the left temporal lobe – a reduction in the volume of white and gray matter compared to the control group (subjects without craniofacial abnormalities). Subsequently, they discovered a pathological enlargement of the superior temporal plane, which correlates with the cognitive deficit [11]. Moreover, they have not identified any link between the structural changes at the superior temporal plane and the auditory deficit present in childhood, concluding that the language disorder is secondary to the cognitive deficit rather than hearing loss. Since the central auditory disorder was relevant for delaying myelinated central nervous system development, especially of the corpus callosum, there is evidence that in children with a cleft palate or cleft lip and cleft palate, there is a risk of developing a hearing disorder due to abnormal P1-N1-P2 responses [11, 12]. In our patient’s case, CAEP should be reevaluated to seek for the appearance of the other components concurrently with cortical maturation.

In our patient, the developmental quotient is lower than 1, suggesting inappropriate age development. A gain score greater than 1 indicates that the rate of progress was higher than expected between the two evaluations (the patient made 12 months of progress during 6 months) [13]. According to the conventional Denver II scale assessment method, both observations scored “suspect”. When using the adapted method, the developmental estimate rate across all four areas showed progress between the two evaluations. In the language field, the patient presented the lowest developmental estimate rate.

Nevertheless, progress was observed after the initiation of auditory-verbal rehabilitation therapy. From the four evaluated domains, the personal-social and language areas show the lowest development estimate rate. We can conclude that language disorder can significantly influence the personal-social domain. These results are consistent with those obtained by Jareean Meizen-Derr et al. in a study which showed that language delays in hearing-impaired children have a direct impact on communication and the social field [14].

The patient also presented developmental delay for both fine and gross motor abilities, which, after the initiation of physical therapy, have significantly improved.

Another issue to be considered is brain plasticity in children with hearing loss and associated disabilities. Brain plasticity may be influenced by the etiology of hearing loss (genetic or environmental factors), and it may have effects on the therapeutic outcomes.

The neurocognitive assessments need to establish a comprehensive framework of measuring outcomes in children with hearing loss, like auditory awareness, speech perception, language and speech development, neurocognitive development, and social skill assessments [15].

## Conclusion

The presented case reveals the importance of using the P1 CAEP biomarker in clinical practice along with a neuropsychological evaluation to assess the maturation of the central auditory pathways and to objectively quantify the results of auditory rehabilitation in children with hearing loss and associated disabilities.

## Conflict of Interest

The authors confirm that there are no conflicts of interest.

## References

[1] Hossain MD, Raghunandhan S, Kameswaran M, Ranjith R (2013). A clinical study of cortical auditory evoked potentials in cochlear implantees. Indian J OtolaryngolHead NeckSurg.

[2] Sharma A, Tobey E, Dorman M (2004). Central auditory maturation and babbling development in infants with cochlear implants. Arch Otolaryngol—Head Neck Surg.

[3] Dorman MF, Sharma A, Gilley P, Martin K, Roland P (2007). Central auditory development: evidence from CAEP measurements in children fit with cochlear implants. J Commun Disord.

[4] Silva LAF, Couto MI, Tsuji RK, Bento RF, de Carvalho AC, Matas CG (2015). Auditory Cortical Maturation in a Child with Cochlear Implant: Analysis of Electrophysiological and Behavioral Measures. Case reports in Otolaryngology.

[5] Sharma A, Dorman MF, Spahr AJ (2002). A sensitive period for the development of the central auditory system in children with cochlear implants: implications for age of implantation. Ear Hear.

[6] Sharma A, Dorman MF (2006). Central auditory development in children with cochlear implants: clinical implications. Adv Otorhinolaryngol.

[7] Sharma A, Glick H (2016). Cross-modal re-organization in clinical populations with hearing loss. Brain Sci.

[8] Sharma A, Glick H, Campbell J, Biever A (2013). Central auditory development in children with hearing impairment: clinical relevance of the P1 CAEP biomarker in children with multiple disabilities. Hearing Balance Commun.

[9] King KA, Campbell J, Sharma A, Martin K, Dorman M, Langran J (2008). The representation of voice onset time in the cortical auditory evoked potentials of young children. Clin Neurophysiol.

[10] Ma X, McPherson B, Ma L (2016). Electrophysiological assessment of auditory processing disorder in children with non-syndromic cleft lip and/or palate. PeerJ.

[11] Nopoulos P, Berg S, VanDemark D, Richman L, Canady J, Andreasen NC (2002). Cognitive dysfunction in adult males with non-syndrome clefts of the lip and/or palate. Neuropsychologia.

[12] Shriver AS, Canady J, Richman L, Andreasen NC, Nopoulos P (2006). Structure and function of the superior temporal plane in adult males with cleft lip and palate: pathologic enlargement with no relationship to childhood hearing deficits. J Child Psychol Psychiatry.

[13] Christine JW, Christine FS, Cary SC, Karen SB (2002). Adaptations of the Denver II scoring system to assess the developmental status of children with medically complex conditions. J Child Health Care.

[14] Meinzen-Derr J, Wiley S, Grether S, Phillips J, Choo D, Hibner J, Barnard H (2014). Functional Communication of Children Who Are Deaf or Hard-of-Hearing. J Dev Behav Pediatr.

[15] Liu X (2016). Current trends in outcome studies for children with hearing loss and the need to establish a comprehensive framework of measuring outcomes in children with hearing loss in China. J Otol.

